# Epigenetic DNA Methylation Mediating *Octopus vulgaris* Early Development: Effect of Essential Fatty Acids Enriched Diet

**DOI:** 10.3389/fphys.2017.00292

**Published:** 2017-05-16

**Authors:** Pablo García-Fernández, Danie García-Souto, Eduardo Almansa, Paloma Morán, Camino Gestal

**Affiliations:** ^1^Aquatic Molecular Pathobiology Group, Instituto de Investigaciones Marinas (Consejo Superior de Investigaciones Científicas)Vigo, Spain; ^2^Departamento de Bioquímica, Xenética e Inmunoloxía, Facultade de Bioloxía, Universidade de VigoVigo, Spain; ^3^Instituto Español de Oceanografía, Centro Oceanográfico de CanariasTenerife, Spain

**Keywords:** aquaculture, DNA methylation, epigenetic, MSAP, *Octopus vulgaris*, paralarvae

## Abstract

The common octopus, *Octopus vulgaris*, is a good candidate for aquaculture but a sustainable production is still unviable due to an almost total mortality during the paralarvae stage. DNA methylation regulates gene expression in the eukaryotic genome, and has been shown to exhibit plasticity throughout *O. vulgaris* life cycle, changing profiles from paralarvae to adult stages. This pattern of methylation could be sensitive to small alterations in nutritional and environmental conditions during the species early development, thus impacting on its health, growth and survival. In this sense, a full understanding of the epigenetic mechanisms operating during *O. vulgaris* development would contribute to optimizing the culture conditions for this species. Paralarvae of *O. vulgaris* were cultured over 28 days post-hatching (dph) using two different *Artemia* sp. based diets: control and a long chain polyunsaturated fatty acids (LC-PUFA) enriched diet. The effect of the diets on the paralarvae DNA global methylation was analyzed by Methyl-Sensitive Amplification Polymorphism (MSAP) and global 5-methylcytosine enzyme-linked immunosorbent assay (ELISA) approaches. The analysis of different methylation states over the time revealed a global demethylation phenomena occurring along *O. vulgaris* early development being directly driven by the age of the paralarvae. A gradual decline in methylated loci (hemimethylated, internal cytosine methylated, and hypermethylated) parallel to a progressive gain in non-methylated (NMT) loci toward the later sampling points was verified regardless of the diet provided and demonstrate a pre-established and well-defined demethylation program during its early development, involving a 20% of the MSAP loci. In addition, a differential behavior between diets was also observed at 20 dph, with a LC-PUFA supplementation effect over the methylation profiles. The present results show significant differences on the paralarvae methylation profiles during its development and a diet effect on these changes. It is characterized by a process of demethylation of the genome at the paralarvae stage and the influence of diet to favor this methylation loss.

## Introduction

One of the cephalopod species with a great potential for intensive aquaculture diversification is the common octopus, *Octopus vulgaris*, since it fulfills many of the criteria for this purpose: a short life cycle, fast growth, good food conversion rate, high reproduction performance, fast adaptation to life in captivity, high nutritional value and market price. Unfortunately, while great efforts have been put into raising octopuses in captivity, the sustainable production of this species is still unviable due to the mass mortality during the planktonic paralarvae phase (Vaz-Pires et al., 2004; Iglesias and Fuentes, 2014). Factors, such as: water quality, temperature, light exposure or nutrition; directly influence on growth, health, and ultimately, survival. In addition, octopus paralarvae from distinct geographical origins could have different behavior under similar culturing conditions, suggesting an interaction between paralarvae adaptability to captivity and genotype (Garrido et al., 2017).

Nutrition has been identified as one of the most critical factors influencing octopus paralarvae viability and survival in captivity conditions (Navarro et al., 2014). In contrast to the standard feeding protocols based on live *Artemia* sp. prey, experimental enriched diets supplemented with long chain polyunsaturated fatty acids (LC-PUFAs) and phospholipids have been shown to be beneficial in terms of growth and survival (Guinot et al., 2013a; Garrido et al., 2016a). LC-PUFAs are known to modulate gene expression by provoking local and global effects over DNA methylation in several organisms (see Burdge and Lillycrop, 2014 for review). DNA methylation, the addition of a methyl group to the C-5 position of a cytosine nucleotide by a DNA methyltransferase (Jin et al., 2011), is the most widely studied epigenetic mechanism. Increasing evidence points out DNA methylation as a mechanism with an important role in gene expression regulation in the eukaryotic genome (Wu and Zhang, 2014). Unprogrammed alterations on the methylation profiles triggered by diet and environmental stressors would lead to aberrant gene expression associated with spurious consequences (Faulk and Dolinoy, 2011). This is particularly true during the early development, when DNA methylation is crucial on genomic reprogramming. These early acquired epigenetic landmarks may affect the phenotype, provoke diseases at the adulthood or cause premature mortality (Faulk and Dolinoy, 2011). Moreover, they may persist throughout the entire animal life and even be transmitted to the following generations by genomic imprinting, conditioning their offspring (Feil and Berger, 2007). Considering the direct impact on gene expression and potential heritability, the analysis of methylation profiles should become a valuable tool, if not essential, for biomonitoring the physiological status of cultured specimens in aquaculture (Moghadam et al., 2015).

Although there is wide evidence demonstrating an interaction between epigenetic mechanisms and environment in mammals, research on invertebrates is still ongoing (Sarda et al., 2012). One of the most representative examples of this phenomenon is found in the honeybee *Apis melifera*, with diet-controlled larvae differentiation into either queen or worker casts positively correlating with their brain methylomes (Lyko et al., 2011). Equally, DNA methylation on the crustacean *Daphnia magna* is labile to exposure to toxic pollutants, conditioning fertility and affecting their future offspring by genome imprinting (Vandegehuchte et al., 2009a,b). DNA methylation research in mollusks is scarce and limited to a few species by using methylation-specific restriction enzymes (Petrović et al., 2009; Díaz-Freije et al., 2014; Sun et al., 2014), quantification by LC-MS (Fneich et al., 2013) and ELISA approaches (Rivière et al., 2013) and genome-wide bisulfite sequencing (Gavery and Roberts, 2013). Gavery and Roberts (2010) confirmed the presence of intragenic CpG island methylation in *Crassostrea gigas*, demonstrating a relationship between predicted methylation status and gene expression. Moreover, the availability of *C. gigas* methylome has exemplified the importance of methylation during the molluscan embryo development and in their adaptability to environmental fluctuations (Gavery and Roberts, 2010; Rivière et al., 2013; Rivière, 2014). All these evidences support a conservative role of methylation in invertebrates, presenting a plastic response to environmental changes and allowing the integration of these signals in the genome, as it happens in vertebrates. In fact, previous studies in *O. vulgaris* have highlighted the important role of DNA methylation during the paralarvae period, when major morphological changes take place (Díaz-Freije et al., 2014).

Under the premise that the paralarvae stage should be sensitive to the environment (including rearing conditions and nutritional aspects), monitoring the methylation status of *O. vulgaris* during this life stage will help assessing the impact of the rearing conditions on their development and, ideally, will predict the later outcome of the culture.

In this sense, we focused our attention on DNA methylation in *O. vulgaris* paralarvae fed two different diets commonly used during the rearing of this life stage. First, the global methylation level in paralarvae was examined using an Enzyme-linked immunosorbent assay (ELISA) and then, methylation status changes, associated with early stages of development and diets, were quantified by means of methylation-sensitive amplified polymorphism (MSAP).

## Materials and methods

### Experimental design and diets

Adult octopuses were captured using artisanal traps in Tenerife coastal waters (Canary Islands, Spain) and maintained as a breeding stock in the facilities of the Oceanographic Centre of the Canary Islands (Spanish Institute of Oceanography). Individuals were kept in 1,000 L tanks (with a maximum density of 10 kg/tank) with water renovation (5 L/min), under dissolved oxygen 100% saturation conditions and low light intensity (400 lx on average). Broodstock were fed *ad libitum* with 50% of frozen crab (*Portunus validus*) and 50% of squid (*Loligo opalescens*) every day. PVC shelters were provided as refuges to enrich the environment and induce natural spawning.

Hatchlings were obtained from spontaneous spawning of one adult octopus female (2 kg) kept in captivity. The female was mature at the moment of the capture and after 2 months was paired with only one male (2.4 kg) which was the main contributor to the offspring. A total of 30,000 paralarvae, 6 replicates of 5,000 paralarvae per tank (10 paralarvae/L) were reared during 28 days in 500 L black fiberglass cylinder-conical tanks. Two fluorescent lights (OSRAM Dulux superstar 36 W/840) were placed above each tank to attain 700 lx focused in the middle of the tank surface with a 12L:12D photoperiod (8:00–20:00). A flow-through seawater system equipped with 20, 5, and 1 μm filter cartridges and UV lamps were used. A water flow per tank of 1 L/min (which promoted over 1.5 renewals/day) was applied from 18:00 to 8:00, removing the excess of *Artemia* sp. through a 500 μm outflow mesh located in the middle of the tank. Two moderated flux aeration stones were placed in front each other in the edges of the tanks. The green-water technique was applied, using 5·10^5^ cell/mL of *Nannochloropsis* sp. (Phytobloom Green Formula®, Olhão, Portugal) that was added to the tanks before turn on light. Temperature and oxygen were daily checked, while nitrite, ammonium and salinity were verified once a week.

Paralarvae were fed with either *Artemia* sp. (Sep-Art BF, INVE Aquaculture, Dendermonde, Belgium) enriched with microalgae *Isochrysis galbana* (T-Iso) and *Nannochloropsis* sp. (control diet from now onwards) or *Artemia* sp. enriched with Marine Lecithin LC 60® (PhosphoTech Laboratoires, Saint Herblain, France) (enriched diet from now onwards). In order to adapt prey size along the experimental period, three *Artemia* sp. sizes (on-growing at different ages) were used along the experimental period: nauplii from 0 to 3 days post-hatching (dph) paralarvae, 4 days old metanauplii from 4 to 11 dph and 8 days old metanauplii from day 12 to 28 dph. The enrichments and on-growing of the *Artemia* sp. was carried out according to Garrido et al. (2017).

### Paralarvae sampling and DNA isolation

Paralarvae growth was assessed at 0, 10, 20, and 28 dph. Dry weight (DW) measurements were individually determined as described by Fuentes et al. (2011). Briefly, paralarvae were euthanized in chilled seawater (−1°C), washed in distilled water, oven dried (110°C, 20 h) and weighted. The specific growth rate (SGR, %DW/day) was expressed as: $SGR\left(\frac{\%DW}{day}\right)=\frac{\left(\ln\;DW_{f}-\ln\;DW_{i}\right)\times 100}{\left(t_{f}-t_{i}\right)}$, where *DW*_*f*_ and *DW*_*i*_ are the *DW* at final time (*t*_*f*_) and initial time (*t*_*i*_), respectively following Garrido et al. (2017) protocol. Dorsal mantle length (DML) measurements were done for each individual with a stereomicroscope (Nikon SMZ-10A. Nikon, Tokyo, Japan) following Villanueva (1995). Survival (*S%*) was assessed at the end of the experiment as: $S\%=100\times \left(\frac{X_{f}}{\left(X_{i}-X_{s}\right)}\right)$, where *X*_*f*_ is the number of alive individuals at the end of experiment, *X*_*i*_ is the initial number of individuals and *X*_*s*_ is the number of sacrificed individuals during the experiment. To detect significant changes in terms of these growth parameters an unpaired *T*-test was performed using the software R.

For DNA methylation analysis, 10 paralarvae were sampled at 0, 10, 20, and 28 dph for each of the two tanks conditions. Larvae were euthanized in chilled seawater (−1°C) and stored in ethanol 100% at −20°C until their analysis. Genomic DNA was individually purified from entire paralarvae using an NZY Tissue gDNA Isolation kit (NZYtech). Subsequently DNA quality and concentration were checked with a Nanodrop-1000 spectrophotometer. DNA extracted samples were adjusted to a final concentration of 100 ng/μL and frozen until use.

All animal experiments were performed in compliance with the Spanish law 65/2013 within the framework of European Union directive on animal welfare (Directive 2010/63/EU) for the protection of animals employed for scientific purposes, following the Guidelines for the care and welfare of cephalopods proposed by Fiorito et al. (2015), and approved by the Ethic Committee of the National Competent Authority.

### Global 5-methylcytosine levels

A global 5-methylcytosine (ELISA) y (5-mC DNA ELISA Kit, ZYMO) was used as a first attempt to measure in an easy and fast way the patterns of the global DNA methylation levels in octopus paralarvae. DNA from three individuals at two different developmental stages including starting developmental point (0 dph) and also 20 dph for both diets were analyzed. Measurements were tested in duplicates, according to the manufacturer instructions. The optical density at 415 nm was determined after 45 min using an iMark™ Microplate Absorbance Reader, (Bio-Rad). The global DNA methylation levels were expressed in percentages as the mean of the two technical replicates and further analyzed using paired *T*-tests in the software R.

### Methylation sensitive amplification polymorphism (MSAP)

A MSAP protocol, adapted from Reyna-López et al. (1997) was applied to 10 paralarvae per sampling point (0, 10, 20, and 28 dph). Briefly, each DNA sample was digested in parallel reactions with either *Eco*RI/*Hpa*II or *Eco*RI/*Msp*I endonucleases. The obtained DNA fragments were ligated with specific adapters and subjected to two consecutive PCR amplification rounds: a first pre-selective PCR, using an *Hpa*II/*Msp*I+T and *Eco*RI+A primer pair was followed by a second selective PCR with 6-FAM labeled *Hpa*II/*Msp*I+TAG and *Hpa*II/*Msp*I+TCC primers. All reactions were run in a GeneAmp PCR system 9,700 (Applied Biosystems). A detailed protocol of the entire procedure is given in Morán and Pérez-Figueroa (2011). Following AFLP reading on an ABI Prism 310 Genetic Analyzer (Applied Biosystems) restriction profiles were scored using the GeneMapper v.3.7 software (Applied Biosystems).

Methylation Sensitive Amplification Polymorphism (MSAP) profiles were assessed from the resulting absence/presence matrix with the R package MSAP (Pérez-Figueroa, 2013). Loci were categorized as non-methylated (NMT) on specimens amplifying bands for both *Hpa*II and *Msp*I digestions, internal cytosine methylated (ICM), or hemimethylated (HMM) if bands were, respectively present only on either *Msp*I or *Hpa*II, or hypermethylated (HPM) whenever both bands were not present for a given specimen. Loci below a 5% error rate threshold and showing < 2 occurrences of each state were systematically excluded from the analysis. Differences among experimental groups were assessed with a multivariate Principal Coordinates Analysis (PCoA) and Analysis of Molecular Variance (AMOVA).

To further assess whether locus-specific methylation on the paralarvae is dependent on diet and/or age, Fisher's exact tests were used to detect candidate loci among the methyl sensitive loci (MSL). After statistical adjustment of the resulting *P*-values according to Benjamini and Hochberg false discovery rate (FDR), only loci showing *P* < 0.05 were selected. Estimates of relationships among selected loci were computed by Gower's Coefficient of Similarity and expressed as Euclidean distances. The resulting matrix was clustered by the average linkage method (UPGMA) and visualized as a heatmap matrix with the R “ComplexHeatmap” package (Gu et al., 2016).

## Results

### Culture data: growth and survival

A total of 30 individual paralarvae were measured at four different sampling points (0, 10, 20, and 28 dph) for each of the two tested diets. Table 1 shows the DW, DML, SGR, and survival ratio parameters of paralarvae fed with control and enriched diet. The individuals at the starting point (0 dph), showed a DW of 0.23 ± 0.03 mg. This DW increased at 10 dph when the paralarvae were fed with the enriched diet, showing a DW significant higher than the control diet. However, at 28 dph the differences in terms of DW were not significant between the tested feeds. The DML increased from 2.15 ± 0.08 mm at hatching to 2.48 ± 0.35 mm at 28 dph in the control diet group. Concerning the enriched diet group, the DML at 28 dph increased reaching 2.71 ± 0.25 mm. The control diet group showed a survival ratio of 1.47 ± 1.28% at the end of the experiment. For enriched diet the survival ratio increased up to reach 11.7 ± 3.41%.

**Table 1:** **Data of growth (reported as DW, dry weight; SGR, specific growth rate; DML, dorsal mantle length) and survival ratio (S) of paralarvae reared with control and enriched diet**.

	**0 dph**	**10 dph**		**20 dph**		**28 dph**	
		**Enriched diet**	**Control diet**	**Enriched diet**	**Control diet**	**Enriched diet**	**Control diet**
DW (mg)	0.23 ± 0.03	0.36 ± 0.06^*^	0.3 ± 0.04^*^	0.37 ± 0.06	0.33 ± 0.06	0.57 ± 0.16	0.50 ± 0.10
SGR (%)		3.59	2.08	2.26	1.74	3.31	2.82
DML (mm)	2.15 ± 0.08	2.52 ± 0.14	2.3 ± 0.14	2.45 ± 0.19	2.25 ± 0.12	2.71 ± 0.25	2.48 ± 0.35
S (%)						11.7 ± 3.41	1.47 ± 1.28

*Differences between control and enriched diet groups at same age were analyzed with an unpaired T-test*.

*DW, Dry weight; SGR, specific growth rate; DML, dorsal mantle length*.

*Data reported with standard deviation*.

* *Indicate significant differences between treatments at the same age (P < 0.05)*.

### Global 5 MeC levels

The 5-mC (ELISA) performed showed that the methylation level of cytosines ranged from 1.21% (minimum values) to 1.24% (maximum value). The methylated cytosine mean level was 1.22 ± 0.07% at the initial stage (0 dph). At 20 dph methylated cytosine mean level was slightly inferior (1.19 ± 0.19%) for larvae fed with the control diet and it slightly increased (1.24 ± 0.25%) for larvae fed with the enriched diet. No differences were found between groups.

### MSAP analyses

The MSAP analyses yielded a total of 297 polymorphic loci when all data from the 70 individuals under study were included (7 groups: 0, 10, 20, and 28 dph for control and enriched diets; 10 individuals per group). Of these, 269 loci were identified as MSL, whereas the remaining 28 ones were non-methyl sensitive (NML). A 100% (28 loci) of the NML were classified as polymorphic whilst the proportion of polymorphic loci reached 70% (188 loci) of the MSL.

**Figure 1:**
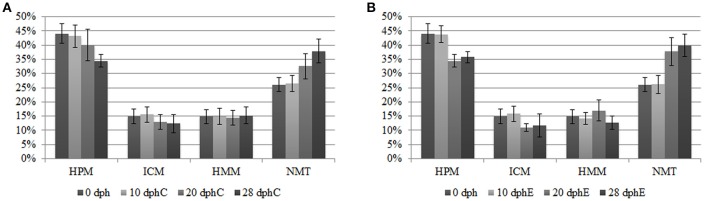
**DNA methylation status of methylation-sensitive loci from MSAP (***HpaII***/***MspI*** comparison) in ***O. vulgaris*** paralarvae (expressed as percentages) measured at 0, 10, 20, and 28 dph**. Methylated loci corresponded to categories: ICM, internal cytosine methylated; HMM, hemimethylated; HPM, hypermethylated, and no methylated loci is referred as NMT, non-methylated. Percentages are referred to the total number of polymorphic loci after the error rate filtering. **(A)** Control diet MSAP status. **(B)** Enriched diet MSAP status.

The analysis of different methylation states over the time revealed a global demethylation phenomena occurring along *O. vulgaris* early development and directly driven by the age of the paralarvae. A gradual decline in methylated categories (HMM, ICM, HPM) parallel to a progressive gain in NMT loci toward the later sampling points was verified regardless of the diet provided (see Figure 1). In fact, the NMT state, representing a 26.12 ± 2.45% of the total at hatching (0 dph), reached maximum values at 28 dph: 37.93 ± 4.08% for the control diet and was even slightly higher, 39.84 ± 3.94%, for the enriched diet group. The AMOVA tests supported a more significant effect of paralarvae development on the methylation profile both in the overall samples (Φ_ST_ = 0.1726, *P* < 0.0001) and separately in the two diets (Control diet: Φ_ST_ = 0.1641, *P* < 0.0001; Enriched diet: Φ_ST_ = 0.2155, *P* < 0.0001). On the other hand, and as expected from the same female and egg spawn paralarvae, AMOVA results on the NML were not statistically significant either when covering all samples (Φ_ST_ = 0.01484, *P* = 0.1633) nor separately for each diet (Control diet: Φ_ST_ = 0.02472, *P* = 0.1617; Enriched diet: Φ_ST_ = −0.008439, *P* = 0.8231), demonstrating the genetic homogeneity among the study samples and thus confirming the validity of the assay.

**Figure 2:**
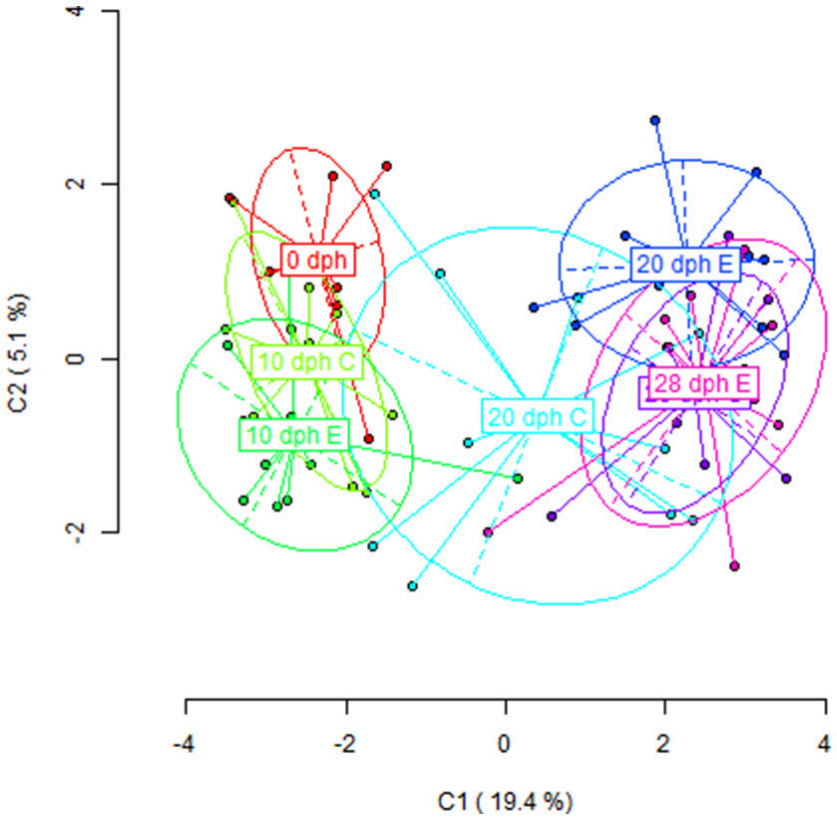
**Results from Principal Coordinate Analysis (PCoA) for the ***HpaII***/***MspI*** comparison in all age-diet groups of ***O. vulgaris*** paralarvae (C, control diet; E, enriched diet)**. The first two axes are shown, indicating the percentage of the global variance explained on the corresponding axis. Points were the representation of the paralarvae individuals and the ellipse delimitates the variance of each group (age + diet).

The PCoA performed with the MSL revealed a grouping of the samples according to this age-driven demethylation process (Figure 2). The first principal component, explaining a 19.4% of the observed variation, clearly discriminates between early (0 dph and 10 dph) and late (28 dph) time-points, with samples from the same period clustering together into relatively compact groups. Nevertheless, a differential behavior between diets was observed at 20 dph. At this age, paralarvae subjected to an enriched feeding diet grouped closer to the later samples (28 dph) than to their equivalents for the control diet. A larger intragroup variability was observed at 20 dph in control paralarvae with regards to their LC-PUFA enriched equivalents. This may be interpreted in terms of a slower and/or random loss of epigenetic marks in the absence of enriched diet supply during this period. More specifically, pairwise comparisons between adjacent sampling times (Table 2) revealed significant differences in DNA methylation profiles only at diet groups the 10–20 dph (Control diet: Φ_ST_ = 0.1411, *P* < 0.0001; Enriched diet: Φ_ST_ = 0.3365, *P* < 0.0001) and 20–28 dph comparisons for both diet groups. Overall, these MSAP results were consistent with a pre-programmed gradual loss of methylation throughout the early development of *O. vulgaris*, having a diet-sensitive period around 20 dph.

**Table 2:** **Comparisons of methylation-sensitive loci distribution for both diets and identification of specific loci**.

	**Φ_ST_ (Pairwise AMOVA)**	***P***	**#Loci (Fisher test)**	**#Loci (Fisher test and FDR < 0.05)**
**CONTROL DIET**				
0–10 dph	0.0051	0.3867	2	0
10–20 dph	0.1411	<0.0001^***^	11	2
20–28 dph	0.0928	0.0022^**^	10	0
**ENRICHED DIET**				
0–10 dph	0.1172	0.0005	5	1
10–20 dph	0.3365	<0.0001^***^	27	15
20–28 dph	0.0637	0.0021^**^	9	0

*Pairwise AMOVA between adjacent ages was analyzing separately for control and enriched diets. Identification of specific loci by Fisher's test and False Discovery Ratio adjust (P < 0.05 and FDR < 0.05)*.

** *Indicate significant differences between treatments at the same age (P < 0.01)*.

*** *Indicate significant differences between treatments at the same age (P < 0.001)*.

*^Φ_ST_^ Analysis of molecular variance Φ_ST_ genetic differentiation (Excoffier et al., 1992)*.

*^#^Number of*.

The statistical analysis of the different loci by the Fisher Exact test allowed the identification of a total of 51 statistically significant loci (Adjusted *P* < 0.05) among experimental groups. These loci presented a differential distribution of its methylation status mainly between diets at the 10–20 dph comparison (Table 2). The result indicates that 15 loci exceeded the FDR cut-off established in the enriched diet compared with the only 2 loci for the same comparison in the control diet. These 2 loci detected in 10–20 dph control diet were also present in the enriched diet group so they were diet independent and correspond to development driven changes. The rest of the loci were identified in the enriched diet group and highlight a specific dietary effect.

**Figure 3:**
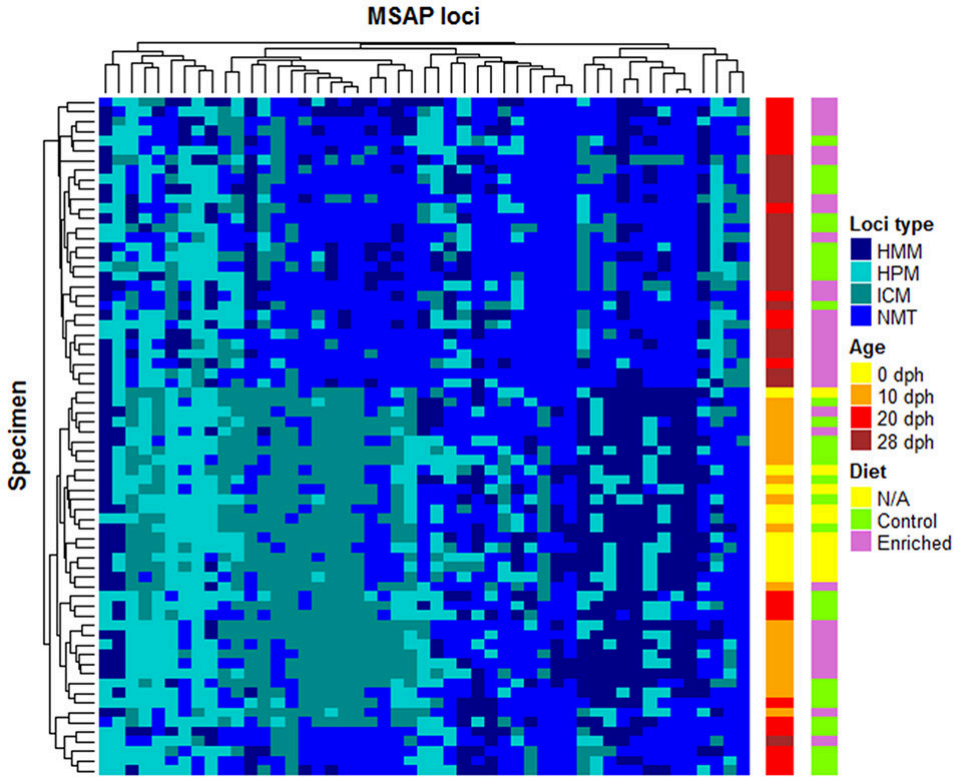
**Heatmap of 51 highly differentially methylated MSL of ***O. vulgaris*** paralarvae**. Specimens (rows) and loci (columns) were clustered by the average linkage method. These 51 MSL are the most highly differentially methylated loci identified by Fisher's test (*P* < 0.05). Diet (N/A for 0 dph because have not received diet) and age are shown at right side of the heatmap.

The representation of these 51 statistically relevant loci in a heatmap split the samples into two major clusters, discriminating between early (0 and 10 dph) and late (28 dph) developmental stages (Figure 3). In a similar way to the previous PCoA on the MSL, samples of 20 dph showed a distinct behavior between diets, with all enriched diet samples grouping with 28 dph paralarvae whilst most control diet samples (9/10) clustered together with 0 and 10 dph samples.

More in detail, the clustering analysis shows that these 51 loci are grouped in 5 clusters (see Figure 3). The first four clusters, ordered from left to right, are characterized by losing methylation with the paralarvae development. In the first one, loci showed mainly transitions from HPM to HMM or NMT from the early developmental paralarvae group to the later one. Loci undergoing complete demethylation from ICM in 0 and 10 dph cluster to 28 dph, but also in 20 dph control samples were found on the second cluster, confirming its intermediate status between early and advanced culturing times are found on the second cluster. The third cluster presents a less clear patterning, although it could group those loci losing its HPM status from 0 dph onwards. There was a fourth cluster of loci which change from HMM status at 0 and 10 dph samples to NMT status on later days. Finally, and despite the loss of methylation is associated with development, the fifth and last cluster of the heatmap contained some loci suffering de novo methylation. The latter showed that, even though there is a general pattern of loss of methylation over this developmental period, there is room for certain positions to undergo de novo methylation, although such events can be considered rare.

## Discussion

Nowadays the only commercial available prey for *O. vulgaris* paralarvae is *Artemia* sp. However, nutrient enrichments are necessary to improve their nutritional quality. In this regard, LC-PUFA and phospholipid enriched supplements are particularly promising, since they boost the paralarvae viability (Guinot et al., 2013b; Garrido et al., 2016a). Our results show a significant improvement of the survival ratio at 28 dph for the *Artemia* sp. enriched with LC-PUFA and phospholipids respect to control diet. Nonetheless, no significant effect was detected in terms of size and DW at 28 dph between both diets despite the higher values found in the DW of LC-PUFA enriched diet. These results appear to be contrary to the positive effect of LC-PUFA described by Garrido et al. (2016a) in the same species, but it must be considered that the last study was a meta-analysis, which found differences integrating many independent experiments whose results were not often significant. In fact, the variability among studies has been also highlighted by the same authors (Garrido et al., 2017). However, a statistically significant increment of the DW was detected at 10 dph in the enriched diet group (see Table 1) suggesting a critical sensitive window where diet could have major effects.

In fish culture, special attention has been taken on lipid and fatty acid requirements due to their essentiality for a correct development and the link between fatty acids and gene expression has already been shown (Tocher, 2010; Xu et al., 2014). However, in *O. vulgaris*, little is known about the molecular basis of the effects of the lipids requirements (Monroig et al., 2013; Reis et al., 2014). For this reason, a better knowledge of octopus genomic regulation mechanisms will be valuable to diet formulation, managing welfare conditions related to culture facilities and to identifying the best conditions to improve breeding programs. To our knowledge, the present research is the first attempt to link diet and methylation in the context of the *O. vulgaris* aquaculture.

The methylation levels herein described for *O. vulgaris* paralarvae using 5-mC (ELISA) (on average 1.2%) are low but in line with those of other mollusks (for example *Biomphalaria glabrate* and *C. gigas*), around 2% (Fneich et al., 2013; Gavery and Roberts, 2013). In contrast to vertebrates, invertebrates present an exceptional variability of how DNA methylation is distributed in their genomes and its function is yet to be completely elucidated. Species of invertebrates like *Caenorhabditis elegans* or dipteran insects like *Drosophila melanogaster* (two model organisms), present an apparent absence of cytosine methylation on their genomes, illustrating that changes in gene expression are independent to DNA methylation (Schübeler, 2015). Nevertheless, DNA methylation in mollusks, such as the oyster *C. gigas*, is likely to vary among life history stages, playing an important role during the embryogenesis of this species and progressively decreasing its levels toward the adult stages, when it no longer exerts a relevant function in the control of gene expression (Rivière, 2014). In fact, previous results on *O. vulgaris* have remarked for an important role of methylation during the earlier development stages (at 1 dph) and having no effect in adults (Díaz-Freije et al., 2014). Our results extend the range of knowledge for this species and demonstrate a pre-established demethylation program during its early development. As methylation levels are low, the 5-mC (ELISA) failed to detect methylation changes in paralarvae either related to age or to diet. However, in this work, the potential of MSAP to analyze methylation patterns in paralarvae have been proved to be highly informative, revealing significant changes in methylation levels, from more methylated DNA of the larvae at hatching to less methylated DNA larvae after 28 days.

Far from being a stochastic phenomenon, our results demonstrate that this loss of methylation follows a well-defined pattern involving a 20% of the MSAP loci, that highlights how some genomic regions are specifically demethylated. Previous research has demonstrated for a pre-defined pattern of active and directed demethylation throughout the early development of vertebrates (Razin and Shemer, 1995; Paranjpe and Veenstra, 2015). The enzymatic machinery behind the methylation process and the changes produced in gene expression are known in detail in vertebrates. Unscheduled alterations provoked by either environmental agents or artificially induced with drugs, such as AZA-5 (5-aza-2′-deoxycytidine) during this period leads to developmental arrest and/or disorders at the adulthood (Rivière et al., 2013). Up to date, octopus DNA methyltransferases have not been described but the presence of cytosine methylation cannot be possible without the enzymatic machinery. In bivalves, these methylation changes correlate with alterations in the expression of *DNMT* orthologue genes, as shown for *C. gigas* and the scallop *Chlamys farreri* (Wang et al., 2014; Lian et al., 2015). In both cases this age-driven demethylation process correlates to low expression levels of the maintenance methyltransferase *DNMT1* and, apparently, undetectable de novo methyltransferases (*DNMT3*). Consequently, this loss of methylation might occur in a passive way, yet preferentially directed toward certain genomic regions.

Although the pattern of methylation loss in octopus paralarvae is mainly age-driven, our results also demonstrate a certain diet influence on the methylation profiles. Indeed, LC-PUFA fed paralarvae showed a premature transition into 28 dph methylation profiles. These results have been demonstrated for a diet-sensitive period in the paralarvae of *O. vulgaris* at around 20 dph and for an effect of LC-PUFA supplementation over the methylation profiles. Despite there are no previous studies correlating this type of dietary supplementation with changes in methylation in invertebrates, the addition of LC-PUFA has shown a global effect over the levels of DNA methylation (Boddicker et al., 2016) and an impact over regulator regions of specific genes (Ma et al., 2016) in vertebrates (well-known in *Mus musculus, Rattus norvegicus* and *Sus scrofa*). The implication of fatty acids on the methylome landmark is well-known but the mechanism behind this effect and their targets are still under study (see Burdge and Lillycrop, 2014 for review). Recent studies have started to elucidate this mechanism, correlating the addition of PUFA and changes over the DNA methyltransferase expression patterns (Huang et al., 2016). Thus, in some cases the dietary input of fatty acids finally acts, trough methylation mechanism, over the regulator regions of clue genes in the metabolic homeostasis. The final consequence is a change in the lipid metabolism. The results obtained by (Xu et al., 2014) have special interest since they show how the dietary input of LC-PUFA induce DNA methylation changes which directly affect the main pathway of biosynthesis of these LC-PUFA. These fatty acids are an essential nutritional requirement during *O. vulgaris* early development (Monroig et al., 2013; Reis et al., 2014). In view of the effect of the diet at 20 dph some similar mechanisms may be running in the octopus paralarvae, which could be sensitive to small alterations in nutritional but also environmental conditions during the paralarvae stage, including the digestive tract functionality, and immune system competence, and thus have a great impact on its health, growth and survival.

In vertebrates, the effect over DNA methylation of a diet rich in LC-PUFA and the impact of that over gene expression modulation have been demonstrated. Also, the knowledge of that kind of mechanisms of regulation has promising uses in aquaculture in terms of improve welfare and performance trough diet design. Nevertheless, it remains unknown if this also applies to invertebrates. For other dietary elements and environmental factors, a plastic response during sensitive periods on the early development has been proposed. This mechanism would act as an adaptation mechanism (Saint-Carlier and Rivière, 2015). For instance, in the honeybee, larvae differentiation into either workers or queens is directly dependent on the diet via global methylation changes (Lyko et al., 2011). In the case of *O. vulgaris* paralarvae our results show a dietary effect at 10–20 dph with no changes on loci methylation at 28 dph between control and enrichment diet. Previous studies have shown that differences in lipid composition between cultured paralarvae and their wild equivalents start to be appreciated at around 10 dph (Navarro and Villanueva, 2003; Garrido et al., 2016b). Such differentiation could be due to alterations in the expression of genes regulating lipid metabolism, mediated, among other mechanisms, by DNA methylation as it has been shown for fishes (Xu et al., 2014).

In summary, we have described the existence of an age-driven predetermined demethylation process during the early development of *O. vulgaris*, with a sensitive period to a LC-PUFA supplemented diet at around 20 dph. Enriched diet paralarvae accelerate its transition into latter developmental methylation profiles and show better survival than those verified in the control diet individuals. Nevertheless, more exhaustive studies have to be performed to check the effect of these changes over gene expression patterns with special interest on those genes involved in fatty acid metabolism.

## Author contributions

PG performed laboratory experiments, statistical analysis and wrote the manuscript. DG collaborated in laboratory experiments, statistical analysis and manuscript writing. EA performed paralarvae culture and sampling. PM supervised DNA methylation analysis and statistical analysis. CG conceived and designed the study and collaborated in the manuscript writing. All authors helped with the draft of the manuscript and approved the final document.

## Funding

This work was funded by “AGL20134910-C02-2R” research Project from the Spanish Ministerio de Economía, Industria y Competitividad. PG (PhD student “Marine Science, Technology and Management–University of Vigo”) thanks Xunta de Galicia for his predoctoral fellowship (“Plan galego de investigación, innovación e crecemento 2011-2015 (Plan I2C)” ref. ED481A-2015/446).

### Conflict of interest statement

The authors declare that the research was conducted in the absence of any commercial or financial relationships that could be construed as a potential conflict of interest.
